# The effects of a four weeks combined resistance training programme on cricket bowling velocity

**DOI:** 10.17159/2078-516X/2021/v33i1a9002

**Published:** 2021-06-10

**Authors:** R Maker, MS Taliep

**Affiliations:** Department of Sport Management, Cape Peninsula University of Technology, Cape Town, South Africa

**Keywords:** sport performance, weighted implement, neuromuscular performance, strength, speed

## Abstract

**Background:**

Despite the importance of resistance training for cricket pace bowlers, there is limited research displaying meaningful improvements in ball release velocity following resistance training.

**Objectives:**

The study aimed at investigating the effects of a four weeks combined resistance training programme on ball release velocity in club cricket pace bowlers.

**Methods:**

Eighteen adult male club level pace bowlers were allocated into a combined resistance training (CRT) group or a traditional cricket training (TR) group. The CRT group (n=9) performed two training sessions a week for four weeks, consisting of a combination of core and lower body strength exercises, plyometric exercises, and weighted implement training. The TR group (n=9) did no resistance training and only bowled with regular weighted cricket balls. Pre-testing/post-testing variables were ball release velocity, bowling accuracy, and upper and lower body neuromuscular performance.

**Results:**

The CRT group significantly increased their ball release velocity by six percent (5.1 km·h^−1^), effect size (ES) =0.65, *p*<0.001) after four weeks of training, while there was no significant difference in the TR group (0.00 km·h^−1^, ES=0.0, *p*=0.674). There was no statistically significant difference in the bowling accuracy and lower body neuromuscular performance for both groups and the upper body neuromuscular performance for the CRT group.

**Conclusion:**

This study provides evidence of a combined resistance training programme that can be used to improve bowling velocity in cricket pace bowlers. This increase in ball velocity was not related to any of the neuromuscular performance variables measured.

The ability to generate high ball speeds has advantages in many reactive ball sports as it gives the opposition less time to respond. In cricket bowling, for example, faster deliveries reduce the batter’s decision-making and execution time of the stroke and increases the chance of the batter being beaten for pace.^[1]^ Surprisingly, there have been few studies investigating the effects of resistance training on ball release velocity in cricket.^[2–4]^ Furthermore, to our knowledge, there have been no studies demonstrating a meaningful increase (>5 km·h^−1^) in ball release velocity (defined as the peak speed between ball release and ball bounce) in cricket pace bowlers following resistance training.

In cricket, fast bowling has been related to both upper body and lower body neuromuscular performance.^[5–6]^ Higher shoulder extension strength was significantly positively correlated to ball release velocity,^[5]^ while neuromuscular performance produced in the lower limbs were associated with increased ball release velocities.^[7]^ During the bowling stride, large ground reaction forces have to be absorbed at front foot landing and powerful deliveries can be generated through leg extension, hip rotation, trunk flexion and shoulder rotation.^[8]^ These studies collectively suggest that resistance training would be beneficial for improving ball release velocity.

Resistance training can broadly be categorised into three types: general, special and specific resistance training. General resistance training, which typically includes the use of free weights, weighted machines, bodyweight exercises and elastic tubing are designed to increase overall strength, and has significantly improved ball release velocity in baseball.^[9]^ Similarly, special resistance training, which is designed to develop power using explosive exercises, such as ballistic and plyometric exercises, has also been shown to improve throwing release velocity in the sport.^[9]^ Specific resistance training provides a training stimulus that mimics the body’s motions and bioenergetic systems that are used in an actual game setting.^[10]^ The use of weighted implement training is a popular example and has shown to improve throwing velocity in baseball.^[11]^ The theory behind the principles of overweight and underweight training can be derived from the force-velocity curve of movement, where resistance training occurs in the motion of the action performed. Training with overweight balls is based on the principle of an overload of force (enhancing strength), whereas training with underweighted balls is based on the principle of an overload of velocity (enhancing speed).^[10]^ Two cricket bowling studies have investigated the use of specific resistance training on ball release velocity.^[3–4]^ Neither of these studies has shown a meaningful increase in ball release velocity. A meaningful increase is defined as the minimum worthwhile velocity of 5 km·h^−1^, which is associated with the smallest change a top order senior club level batter would notice.^[3]^

The combination of general, special and specific resistance (combined) would potentially produce better results. Thus a combination of resistance training appears to improve a wider variety of athletic performance.^[12]^ In cricket bowling, a combined resistance training programme was investigated on recreational bowlers over eight weeks.^[2]^ The results revealed a 3 km·h^−1^ increase in ball release velocity; however, there was also a significant decrease in bowling accuracy. Bowling accuracy can be defined as the ability of a bowler to hit a predetermined vertical target, placed at the position of the cricket stumps.^[2,13]^ Cricket pace bowling performance is dependent on both speed and accuracy. Despite the increase in ball release velocity, it would not relate to improved all-round bowling performance because of the loss of bowling accuracy. The decline in bowling accuracy could be due to the relative high mass of the heavier ball used during training (60% and 92% respectively). When weighted balls are too heavy, it can negatively affect bowling accuracy and disrupt the bowling pattern.^[4]^ This leads to inconsistent delivery release points and decreased bowling accuracy. If this is the case, it might be corrected by using underweight alongside overweight balls or decreasing the mass of the overweight balls utilised.

At the elite level, cricket players have access to physical trainers and physical training resources, and their time can be dedicated to performance enhancement. However, at an amateur (recreational) club level, this will probably not be the case. Recreational players are less likely to have the time available to do extra training at a gym because they may have a full-time job or are full time students, perhaps even with family responsibilities. Others may have the financial resources to join a gym. Therefore, to optimise training for amateur club players, utilising an effective resistance training programme that is cost effective in conjunction with their regular net practice sessions would be ideal. For this reason, combined resistance training could potentially produce the best results in the shortest period. Previous pace bowling research using a combined resistance training programme showed a small increase in ball release velocity accompanied by a decrease in accuracy.^[2]^ Furthermore, the weighted implement training used in this study did not incorporate underweight balls. We therefore hypothesised that by utilising a combined resistance training and including relatively low to moderate over-and underweighted implements, it would increase the ball release velocity without affecting bowling accuracy, compared to participants that only perform traditional cricket training.

## Methods

### Participants

Eighteen male, club level (1^st^ and 2^nd^ division) cricketers were recruited for the study (Table 1). All the participants had been injury-free for at least one month prior to the start of the study. A quasi-experimental design was employed. The participants were divided into a combined resistance training (CRT) group (n=9) and a traditional cricket training (TR) (n=9) group. Selection of players into the CRT group was based on the coaches’ and players’ willingness to allow the training to occur in conjunction with their regular training. Furthermore, the TR and CRT groups were recruited such that no two players from different groups would see the other train. Written informed consent was obtained from all participants prior to commencement of the study. The study had been approved by the authors’ institutional ethics review board (No. 2016FBREC359).

The four-week programme consisted of a combination of general, special (Table 2), and specific resistance training (Table 3). The training programme coincided with the players’ regular cricket net training, which occurred twice a week. The weighted implement bowling occurred first, and thereafter, the general and special resistance training was completed. Training sessions were separated by approximately 48 hours between sessions.

General and special resistance exercises were based on prescribed exercises for cricketers and in line with strength and conditioning needs and training recommendations for pace bowlers.^[14–15]^ The sport-specific bowling protocol utilised overweight, underweight and regular weighted cricket balls (specific resistance) (Table 3). All overweight and underweight balls were professionally manufactured and cost the same as a regular weighted ball. Participants in the TR group only bowled with regular weighted cricket balls (bowled the same number of balls as the CRT group) and were instructed not to perform any resistance training during the four weeks of the study. The ball sequence, mass and number of balls bowled per session is presented in Table 3. All participants played and bowled in one match per week (on a Saturday) during the trial period. The bowling volume during matches was not controlled.

### Measures

The testing variables were ball release velocity, accuracy, and upper and lower body neuromuscular performance. Participants were tested at baseline after the second and fourth week of training. The participants trained on Tuesday and Thursday afternoons and played matches on Saturdays. Testing either took place on a Wednesday or Monday afternoon, allowing a recovery period prior to testing. Before each testing session, participants were required to complete a warm-up. The warm-up started with 20 m shuttle runs of progressive intensity and was followed by both dynamic and static stretching. The warm-up was individualised (each participant performed their own exercises that they usually perform prior to competition) but was standardised within participants between testing sessions. After this general warm-up, the participants bowled several deliveries beginning with light-intensity and progressing to high-intensity. The testing started once the players were satisfied that they were appropriately warmed up.

Participants were instructed to bowl six good-length deliveries (where the ball lands approximately 5 m from the batting crease) at their fastest speed while aiming to hit the top of the off stump after the bounce. However, no formal rating of perceived exertion was measured. The ball release velocity was measured using the Stalker Pro II radar gun, which was placed on a tripod at the end of the participant’s run-up, behind the stumps. There is a maximum estimated error of 0.4% in ball release velocity when the radar gun is positioned behind the stumps (Stalker Pro II; USA). The mean ball release velocity of six deliveries was recorded.

Bowling accuracy was measured using a vertical target grid system.^[2,13]^ Points were awarded according to where the target was hit, with a maximum of 100 points for a delivery hitting the equivalent of the top of the off-stump. Locations further away from the target received less points. The location of where the ball contacted the target grid was recorded with a high-speed video camera at 210 frames per second (Casio Exilim EX-FH20). Bowling accuracy was calculated as the mean points of the six bowled with a regular weighted ball (156 g).

Upper body neuromuscular performance was measured using the seated medicine ball throw test. Seated participants were instructed to push a 3 kg medicine ball from the chest up at approximately a 45° angle as forcefully as possible. No countermovement was allowed. Participants were permitted three warm-up throws. The furthest distance of three attempts was recorded. Lower body neuromuscular performance was measured by a countermovement jump test. The countermovement jump was conducted using the Vertec Jump (USA; California). Participants were allowed three warm-up throws. Thereafter, the highest of the three jumps was used for analysis. To increase adherence to the training protocol, the players’ coaches were familiarised with the exercises and the training protocol to assist and monitor the players. The investigator randomly visited the training sessions to further monitor the training. An attendance adherence of 96% was reported.

### Statistical analysis

Statistical power for ball release velocity was conducted through a general linear model, repeated-measures analysis of variance, within-between factors (F test). A minimum of 18 participants were required for this study (nine per group). The alpha error probability was set to 0.05 and the power (1-β error probability) was 0.96 for the two groups with three repeated measurements (baseline, two weeks and four weeks). All data were first checked for normality (Shapiro-Wilk test), kurtosis, and skewness. All variables were approximately normally distributed. An independent samples t-test was conducted to ascertain if there were any between-group differences in participant characteristics at the pre-test period. The measurements of the CRT and TR groups were compared using a general linear model, repeated measures (within-subjects) and the confidence interval were adjusted using the Bonferroni correction. Effect sizes (ES) were reported by the Hedge’s *g* statistic. The precision of mean differences was expressed with 95% confidence limits (95% CLs). Qualitative descriptors of standardised ES using Hedge’s g were assessed using the criteria: trivial, less than 0.2, small 0.2–0.49, moderate 0.5–0.79, and large 0.8. Correlation between ball release velocity and the other testing variables were pooled for both groups and reported using the Pearson r statistic. Statistical significance for all tests was considered when *p*<0.05.

## Results

**Fig. 1:**
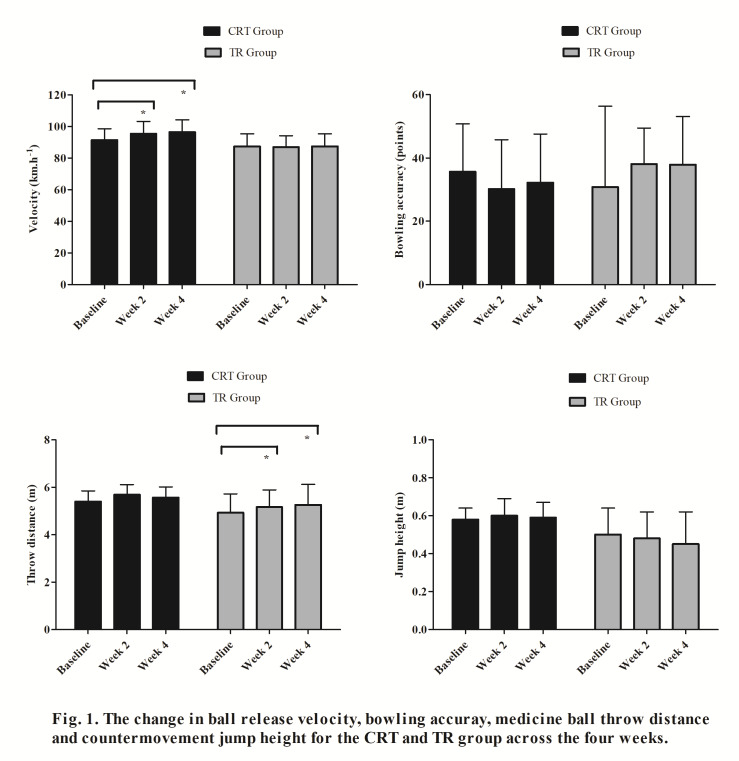
The change in ball release velocity (A), bowling accuracy (B), medicine ball throw distance (C) and countermovement jump height (D) for the CRT and TR groups across the four weeks. CRT, combined resistance training group represented by black bars; TR, traditional cricket training group represented by grey bars.

There were no significant differences in the participant characteristics between groups at baseline (Table 1). The mean ball release velocity differed significantly after four weeks of training [F _(2, 7)_ =51.5, *p*<0.001]. The ball release velocity increased in the CRT group by an average of 4.2 km·h^−1^ (95% [1.6, 6.7], ES=0.52, *p*=0.003) after two weeks and a total increase of 6% (5.1 km·h^−1^, 95% [3.7, 6.6], ES=0.68, *p*<0.001) after four weeks of training (Fig. 1a). This increase in ball release velocity was outside of the error measurement for the radar gun, thus representing a true change in velocity by the bowlers. There was no significant difference in ball release velocity between two weeks and four weeks of training (0.97 km·h^−1^, 95% [−1.4, 3.3], ES=0.12, *p*=0.756) (Fig. 1a). There were no significant differences in the ball release velocity in the TR group across the four weeks [F _(2, 7)_ =0.42, ES=0.0, *p*=0.674] (Fig. 1a). There was no significant difference between accuracy across the four weeks for both the CRT [F _(2, 7)_ =0.72, ES=0.22 *p*=0.520] and TR groups [F _(2, 7)_ =0.35, ES=0.32, *p*=0.718] (Fig. 1b).

There was a non-significant increase in the throw distance across the four weeks [F (_2, 7_) = 2.2, ES=0.36, *p*=0.179] in the CRT group (Fig. 1c). The TR group showed a significant increase in the throw distance across the four weeks ([F _(2, 7)_ = 7.6, *p*=0.018]. The mean throw distance significantly increased between baseline and two weeks (0.24 m, 95% [0.02, 0.46], ES=0.32, *p*=0.032) and between baseline and four weeks (0.33 m, 95% [0.08, 0.59], ES=0.40, *p*=0.013) (Fig. 1c). There was no significant difference in jump height across the four weeks for both the CRT [F _(2, 7)_ =0.42, ES=0.15, *p*=0.762] and TR groups [F _(2, 7)_ = 1.5, ES=0.25, *p*=0.287] (Fig. 1d).

There was a statistically significant positive correlation between the countermovement jump height and ball release velocity (r=0.681, p=0.003). There was no statistically significant correlation between ball release velocity and medicine ball throw distance (r=0.238, p=0.342) or bowling accuracy (r=−0.400, p=0.100).

## Discussion

This study supports the hypothesis that a combined resistance training programme would increase ball release velocity in recreational cricket pace bowlers without a statistically significant decrease in bowling accuracy. In comparison with previous resistance training studies in other sports^[9,11]^, the significant 6% increase in ball release velocity in four weeks is one of the largest increases in ball release velocity reported for such a short period of time, even when training recreational (club) participants.

The non-statistically significant difference in both upper and lower body neuromuscular performance within the CRT group suggests that the observed increase in the ball release velocity in the CRT group was likely because of the weighted implement training. The advantage of performing weighted implement training is that it is a cricket bowling-specific method and optimises the full arm swing characteristic of cricket pace bowling; and strength gains are in accordance with the bowling movement. The increase in ball release velocity is likely due to a development of power in the arm, and shoulder complex and occurs through similar neural recruitment patterns during the bowling motion. The percentage weighted implements used in this study (10–15%) appears to be beneficial to increase ball release velocity. Previous cricket studies that have failed to show a meaningful increase in ball release velocity in cricket could be related to the percentage weighted implements used.^[2–4]^ The weighted implement used in these studies may either have been too low (3% to 16%) over a 10-week period ^[3]^ or too high (46% to 137%) ^[4]^ and (60% to 92%) ^[2]^ over an 8-week period.

There were non-significant variations in the mean age between the CRT and the TR groups. The effect of age is unlikely to influence the trial results because resistance training responses have been found to be similar for adults irrespective of their age.^[16]^ The CRT group also had a non-significant lower resistance training age compared the TR group. These participants would potentially respond quickly to resistance training. However, previous studies that used relatively inexperienced resistance training participants^[2]^ or untrained participants^[9]^ reported small increases (3.7% and 1.7% respectively) in ball release velocity.

The increase in the ball release velocity observed in the CRT group was not at the expense of accuracy, which is a scenario frequently experienced in other studies of this nature.^[2–3]^ The speed and accuracy relationship are of the utmost importance as a delivery with high velocity has little value if it is delivered with poor accuracy. Therefore, the improvement in ball release velocity would positively affect its members’ bowling performance. Previous studies reported a decrease in bowling accuracy.^[2–3]^ The reason for the decrease in bowling accuracy by Petersen et al.^[3]^ could be related to the use of a target zone on a pitch. This system does not take the line of the delivery into account, rendering the validity of the accuracy measure open to question. The reason for the decrease in accuracy in the study by Feros et al.^[2]^ could be the large weighted implements used which could affect the bowling pattern, leading to inconsistent delivery release points and poorer bowling accuracy.^[2,4]^

There was no significant increase in upper neuromuscular performance following training. These findings are similar to those of Feros et al.^[6]^ who found that the increase in ball release velocity was not associated with an increase in one RM pull up strength in cricket pace bowling. Furthermore, the authors observed a weak correlation between the medicine ball toss distance and ball release velocity. Feros et al.^[6]^ similarly found a non-significant correlation between upper body neuromuscular performance and ball release velocity. Both the CRT and the TR groups showed an increase in throw distance after four weeks; however, only the TR was statistically significant (*p*=0.179 and *p*=0.032 respectively). Furthermore, the magnitude of the effects was small for both the CRT and TR groups (effect size of 0.36 and 0.40 respectively). The most likely explanation for this is that there was a learning effect. Beckham et al.^[17]^ discusses the importance of familiarisation for the seated medicine ball throw and highlights that several throws are required prior to testing. In this present study, participants were only allowed three warm-up attempts prior to performing the test. The CRT group and TR group increased by 29 cm and 24 cm respectively after two weeks. These authors therefore suggest that the increase in throwing distance is as a result of familiarisation to the test. The limitation of this testing protocol is that the throw scores were not monitored to determine if it was stabilised prior to testing.

Lower body neuromuscular performance did not significantly change across the intervention period. These results are similar to handball that reported no statistical significant change in lower body neuromuscular performance measured by the countermovement jump test, despite there being a significant statistical increase in throwing ball release velocity.^[18]^ There was; however, a significant positive correlation between the countermovement jump height and the ball release velocity, indicating that the test utilised can be used as measure of neuromuscular performance associated with pace ball release velocity. Previous research differed on the relationship between the countermovement jump test and ball release velocity. Pyne et al.^[7]^ found a significant positive correlation, while Feros et al.^[6]^ found a poor non-significant correlation.

### Limitations

There are a few limitations to consider with regard to the findings of this study. The limitation of combined resistance training is that there are a few independent variables that could have contributed to the increase in ball release velocity. Despite our speculation that the weighted implement training largely contributed to the increase in ball release velocity, the exact contribution of the general, special and specific resistance training is unknown.

A further limitation is that the trial was performed in-season and the influence of technical coaching on bowling biomechanics was not controlled for. Biomechanical factors have been associated with pace bowling ^[19]^; however, the influence of biomechanical training on ball release velocity still needs to be confirmed, particularly over a short period of four weeks. Although all the participants reported that they played and bowled in matches during the study’s trial period, the volume of this bowling could not be controlled for due to the competitive nature of the game. It is; however, unlikely that if there were differences in the bowling volume that it would contribute to increased ball speeds because the participants were bowling with regular weighted balls and the magnitude of the difference would be relatively small compared to other studies where volume influenced ball release velocity.^[20]^

Another limitation is that the total training load between the CRT and TR groups was not controlled for which could result in metabolic adaptations in the CRT group. This study did not measure the batter’s perception of the bowler’s performance and therefore the practical application remains in question. Future studies should investigate if a meaningful increase in ball release velocity is related to increased perceived difficulty in the batter’s response or a decrease in batting performance.

## Conclusion

This study provides evidence that the use of combined resistance training improved ball release velocity without having a detrimental effect on the bowling accuracy of club level pace bowlers. The training programme provides coaches and trainers with a cost-effective (only requirement is weighted balls and a 3 kg medicine ball) and time-efficient (can be incorporated with regularly net practice) solution to improve fast bowling performance at a club level. We recommend that approximately 40 balls per week be delivered with 10–15% weighted implement to improve fast bowling speed.

## Figures and Tables

**Table 1:** Participant characteristics and testing variables at baseline

	CRT group (n = 9)	TR group (n = 9)	*p* – value
**Participant characteristics**			
Height (m)	1.78 ± 0.1	1.73 ± 0.1	0.231
Mass (kg)	73.0 ± 8.3	77.0 ± 21.4	0.605
Age (years)	22.6 ± 5.1	28.3 ± 7.7	0.078
Resistance training experience (years)	0.8 ± 1.1	3.3 ± 4.6	0.131
Pace bowling experience (years)	10.3 ± 5.5	12.0 ± 5.2	0.518
**Testing variables**			
Ball velocity (km.h^−1^)	91 ± 7	87 ± 8	0.301
Bowling accuracy (points)	36 ± 15	31 ± 26	0.628
Throw distance (m)	5.4 ± 0.5	4.9 ± 0.8	0.141
Jump height (cm)	0.58 ± 0.1	0.49 ± 0.1	0.105

Data are expressed as mean ± SD. CRT, combined resistance training group; TR, traditional cricket training group.

**Table 2:** Summary of the four-week general and specific resistance training programme. Sets were separated by 1 minute

Week	Exercise name	Type of resistance training	Reps x Sets
1–2	Bodyweight squats	General	10 x 2
	Bilateral hip raises	General	10 x 2
	Bodyweight split squats	General	6 x 2
	Chest passes	Special	6 x 2
	Recoiled overhead slams	Special	6 x 2
	Standing side tosses	Special	6 x 2
	Recoiled rotational shot puts	Special	6 x 2

3–4	Bodyweight squats	General	12 x 2
	Bodyweight split squats	General	8 x 2
	Bilateral hip raises	General	12 x 2
	Chest passes	Special	8 x 2
	Recoiled overhead slams	Special	8 x 2
	Hop back and throws	Special	8 x 2
	Step behind and throws	Special	8 x 2

**Table 3:** Ball mass and bowling sequence for the CRT and TR group

Week	Ball sequence and mass (CRT)	Ball sequence and mass (TR)	Balls bowled per session for each group
1–2	4 x 156 g (0%)	4 x 156 g (0%)	24 (4 overs)
	8 x 172 g (10%)	8 x 156 g (0%)	
	8 x 140 g (−10%)	8 x 156 g (0%)	
	4 x 156 g (0%)	4 x 156 g (0%)	

3–4	5 x 156 g (0%)	5 x 156 g (0%)	30 (5 overs)
	10 x 179 g (15%)	10 x 156 g (0%)	
	10 x 133 g (−15%)	10 x 156 g (0%)	
	5 x 156 g (0%)	5 x 156 g (0%)	

Overweight and underweight balls were rounded off to the nearest gram. Ball mass above or below the normal mass are indicated as a percentage. CRT, combined resistance training group; TR, traditional cricket training group.
